# Everybody's Page

**Published:** 1890-12-13

**Authors:** 


### EVERYBODY'S PAGE.

THE prevalence of consumption amongst troops in Europe has been shown by statistics to be most severe among the English, and least severe among the French.

THAT most grievous of our minor ills, the toothache, may be sometimes cured, and usually alleviated, by a weak galvanic current which can be generated by placing a silver coin on one side of the mouth and a piece of zinc on the other. To increase the effect, rinsing the mouth with acidulated water will be found efficacious.

IF Mr. Elliott's invention for freeing our cities and towns from smoke by a process of absorbing it in water accomplishes all that is claimed on its behalf, he will rank among the greatest benefactors of mankind. London and other large towns freed from the solid particles of smoke, and the noxious gases and sulphurous vapours, will become a comparative paradise.

THERE was, a short time ago, quite an epidemic in London of attempts to commit suicide by drowning, possibly owing to the warmth of the water during the muggy weather. But the change in the temperature of the atmosphere and water had a remarkably beneficial effect on one gentleman of suicidal tendencies, who was impelled by the cold to make the promptest endeavours to swim to shore. The cold water cure is as simple as it is efficacious.

WITH reference to an appeal which is made to the British public for aid to maintain a leper hospital in Japan, which has been established by a French priest, a correspondent in the *Times* pertinently points out that the Japanese are not in want of funds or foreign medical aid in order to carry on their hospitals, and that we might as reasonably appeal to Japan for help. The fact is, England is looked on as fair game by every begging apostle of charity throughout the world.

PUBLIC dissatisfaction with the policy of the School Board for London is taking tangible shape. Extravagance and waste in expenditure have reached such a pitch that the "public worm," or British ratepayer, roused by apprehension for the future, is beginning to turn. It was the unanimously expressed opinion of the recent meeting of delegates appointed by various vestries and district boards in the administrative County of London that the School Board has not kept the spirit of the Act which called it into existence, as that Act was passed to provide for the children of the very poor a plain, elementary education, while the Board has steadily and recklessly increased the number of advanced subjects to be taught, and thus encouraged the attendance of children whose parents are well able to afford a proper education for them other than by the assistance of the rates. Though the Board was not meant to stand still, it was not created for the purpose of waste.

DR. KERR, Medical Missionary of Canton, has not let the grass grow under his feet during the past thirty-six years, if report be true. He has treated over 520,000 patients, he has prepared twenty-seven medical and surgical books, and has trained one hundred medical assistants.

AN old Scotchman was very ill indeed, and a kind-hearted lady in his parish sent him soup daily. He got better after a while, and the minister called and told him how grateful he ought to be to Providence for his recovery. "Hech! sir," said the old man, "I dinna want to wrang the Almighty, but I think it was Mrs. Allison's good soup that did it"!

THE person who is troubled by obesity might, it is affirmed by Dr. Griffith in the *Indiana Medical Journal*, reduce himself to slim and graceful proportions if he would but take pills made from extract of "poke-berries." So here is an opportunity for him who knows a "poke-berry" when he sees it. Our knowledge of botany, we regret to say, falls short of this interesting point.

IT will surprise nobody to learn that the Americans, with their natural facility for outdoing other nations, surpass the inhabitants of "Yarrup" in even the matter of longevity. In their highly-favoured country the average annual deaths in every thousand are eighteen, while in England the rate is twenty to a thousand, the returns from Germany being still more dismal, namely, twenty-six out of a thousand.

THE new Society for the Encouragement and Preservation of Indian Art deserves the fullest support. It is a matter of common notoriety that the intrinsic qualities of Indian art are in peril of being lost to the world. Originality of design amongst the natives is rapidly disappearing, and giving place to the copying of incongruous Western designs. All lovers of art will rejoice if the Society succeeds in staying the advance of the deteriorating influence which the example of meretricious Western taste has exercised over Indian artists.

THE difficulties which surround the question of how to deal satisfactorily with insanitary dwellings are now being exemplified in Bethnal Green. Between fifty and sixty houses in the district of St. Matthew, Bethnal Green, have been certified by the sanitary inspectors as unfit for human habitation. The owners, though served with notice to put the tenements in habitable repair, have failed to do so. It now appears that there is no probability of being able to provide the inhabitants of the closed houses with other dwellings. In such cases this is always a difficulty, but in this case it is not the only one. The Housing of the Working Classes Act is silent as to how soon an order for the closing of such tenements is to take effect. In these days of hurry it is hopeless to expect Acts of Parliament, or apparently anything else, to be framed efficiently. The great aim seems to get a thing done, no matter how badly, provided it is done.

